# Reconstructive surgery in immunocompromised patients: evaluation and therapy

**DOI:** 10.3205/iprs000077

**Published:** 2015-12-15

**Authors:** Sebastian E. Dunda, Ahmet Bozkurt, Norbert Pallua, Björn Dirk Krapohl

**Affiliations:** 1 Department of Plastic Surgery and Hand Surgery, St. Marien-Hospital Berlin, Germany; 2 Department of Plastic and Aesthetic Surgery, Hand Surgery, Markus-Hospital Frankfurt, Germany; 3 Department of Plastic Surgery, Reconstructive and Hand Surgery, Burn Center, RWTH University Hospital Aachen, Germany; 4 Center for Musculoskeletal Surgery, Charité – Medical University of Berlin, Germany

**Keywords:** reconstructive surgery, plastic surgery, immunocompromised patients

## Abstract

**Background:** An increasing number of patients undergoing reconstructive surgery are immunocompromised due to different reasons and different medical treatments. Some of the used immunosuppressive drugs may affect the process of wound healing and thereby, impair the long-term success of surgical treatment. Therefore, this retrospective analysis aimed at the evaluation of the perioperative treatment and surgical outcome of immunocompromised patients undergoing different reconstructive procedures.

**Methods:** A retrospective review was performed of 8 immunocompromised patients with different primary diseases who needed reconstructive surgery: 2 patients with non-Hodgkin lymphoma, 1 patient with an acute myeloid leukemia, 1 patient with colitis ulcerosa, 1 patient with liver cirrhosis, 1 patient with chronic polyarthritis, and 2 patients with malignant melanoma.

**Results:** In 7 of our 8 presented cases, multiple operations with wound debridements have been necessary to optimize the granulation of the wound bed before reconstructive surgery. 3 out of these 7 patients required further operations due to wound dehiscence or necrosis, with 2 of them as a result of increased immunosuppressive therapy. 5 out of 8 patients needed no further surgical treatment.

**Conclusions:** Both the perioperative drug therapy and the reconstructive surgery concept need to be determined carefully in each individual case of the immunocompromised patients. Thus, the appropriate point in time of operation to achieve the best possible wound healing as well as the complexity of the procedure will require the consideration of a ‘less is more’ strategy in selected cases.

## Zusammenfassung

**Hintergrund:** Die Zahl immunsupprimierter Patienten in der rekonstruktiven Chirurgie steigt stetig. Einige Immunsuppressiva verzögern den Prozess der Wundheilung und haben somit einen negativen Einfluss auf das Ergebnis nach chirurgischen Interventionen. Daher planten wir diese retrospektive Analyse mit dem Ziel, den Einfluss von Immunsuppression auf das Ergebnis nach chirurgischen Interventionen bei Patienten, bei denen verschiedene rekonstruktive Eingriffe indiziert waren, darzustellen.

**Methoden:** Eine retrospektive Evaluation erfolgte bei 8 immunsupprimierten Patienten, die bei unterschiedlichen Primärerkrankungen rekonstruktive Eingriffe erforderten: 2 Patienten litten an Non-Hodgkin-Lymphomen, 1 Patient an akuter myeloischer Leukämie, 1 Patient an Colitis ulcerose, 1 Patient an Leberzirrhose, 1 Patient an chronischer Polyarthritis und 2 Patienten an einem malignen Melanom.

**Ergebnisse:** Bei 7 der 8 Patienten waren multiple Operationen mit Wunddebridements erforderlich, um die Granulation des Wundbetts zu konditionieren, bevor ein rekonstruktiver Eingriff erfolgversprechend durchgeführt werden konnte. 3 dieser 7 Patienten benötigten danach weitere Eingriffe wegen Wunddehiszenz und Gewebsnekrose, bei 2 Patienten aufgrund von erhöhter immunsuppressiver Therapie. Insgesamt waren bei 5 der 8 Patienten keine Folgeeingriffe nach dem eigentlichen rekonstruktiven Eingriff erforderlich.

**Schlussfolgerung:** Sowohl die perioperative immunsuppressive Therapie als auch das rekonstruktiv-chirurgische Konzept bedürfen der sorgfältigen Evaluation bei jedem Einzelfall immunsupprimierter Patienten. Somit ist der richtige Zeitpunkt des rekonstruktiven Eingriffs mit der besten Aussicht auf Wundheilung wie auch die Abstimmung des Ausmaßes des Eingriffs im Sinne einer „Weniger-ist-mehr-Strategie“ entscheidend für das chirurgische Ergebnis in selektierten Einzelfällen.

## Introduction

In the last decades, plastic and reconstructive microsurgery improved with the development of microsurgical equipment and with new strategies in reconstructive flap design as well as additonal wound therapy techniques. However, with increased life expectancy and the concurrent development of immunosuppressive agents and their possible usage in a variety of diseases, the surgical treatment of immunocompromised patients has become a frequent clinical situation confronting the surgeon with new perioperative frontiers, mainly impaired wound healing and increased risk of wound infection [1], [2], [3], [4], [5], [6], [7], [8].

In the different fields of surgery, various strategies in the perioperative treatment of immunocompromised patients have been worked out and are more or less utilized depending on the concept of the surgeon. In 1988, Cohen et al. presented a perioperative drug therapy protocol for immunosuppressed patients following organ-transplant who needed reconstructive surgery [6]. They reported that 13 of 15 transplanted patients healed primarily without any complications and only two patients required a second operation. Qi et al. demonstrated their experience with reconstructive surgery in 17 patients after solid organ transplantation with solely good postoperative results [4]. Furthermore, they demonstrated that there is no need for transplanted patients to change or even to stop the immunosuppressive drug therapy prior to operation. Most of these reports are focused on renal-transplant patients who needed tumor excision and reconstruction in the head and neck area as a result of their immunosuppressive therapy [3], [7], [8], [9]. 

However, there are only few numbers of reported cases of immunocompromised patients who needed immediate reconstructive surgery following severe soft tissue loss for example after local infections [7], [10], [11]. These local infections may raise from small trauma in the tumor patient, from failed puncture with perivascular necrosis in the immunocompromised patient and for many other reasons. The perioperative treatment of this growing patient group is an exceptional challenge requiring not only surgical skills or perioperative medical treatment but also an individual, sometimes interdisciplinary concept in order to perform the adequate procedure. Therefore, in this retrospective study, we evaluated the surgical treatment and the perioperative therapy of immunocompromised patients without organ transplantation who required reconstructive surgery.

## Material and methods

We identified eight immunocompromised patients with different medical histories, who were treated in the department of plastic surgery including 2 patients with a non-Hodgkin lymphoma, 1 patient with acute myeloid leukemia, 1 patient with colitis ulcerosa, 1 patient with liver cirrhosis, 1 patient with chronic polyarthritis and 2 patients with malignant melanoma (Table 1 (Tab. 1)).

Most of these patients were referred to us from other departments with the exception of two patients diagnosed with hand phlegmons, who presented directly to our emergency department. However, in all presented cases, the patients were immunocompromised due to various reasons and were also undergoing medical immunosuppressive therapy at the time of admission.

## Patients

### Case 1

**Figure 1:**
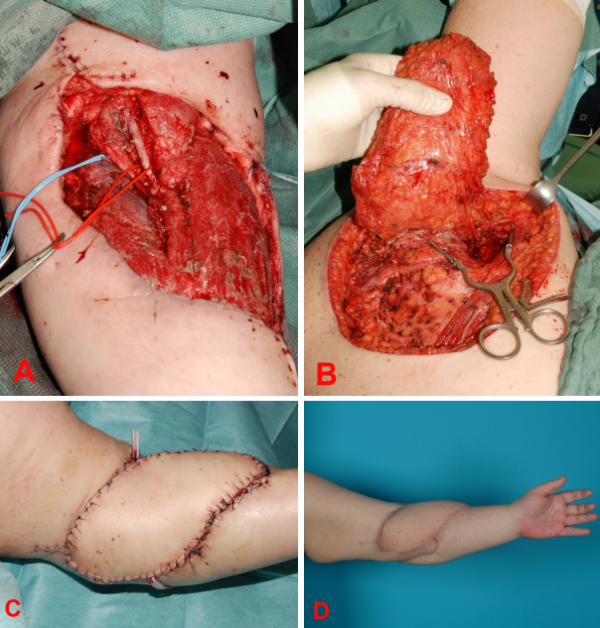
A 50-year-old female with the intraoperative defect on her left upper limb after a doxorubicin extravasate (A); the elevated parascapular flap (B); result 2 days after surgery (C) and follow up after 3 months (D).

A 50-year-old female diagnosed with B-cell non-Hodgkin lymphoma undergoing chemotherapy with rituximab, cyclophosphamide, vincristine, doxorubicin and high-dosage prednisone was presented to our department with a massive and painful swelling in the cubital area of her left arm after an extravasate with doxorubicin. As an initial surgical treatment, we incised the wound following local debridement. Within 7 days of appropriate treatment with daily wound lavage and antibiotic therapy, the affected wound area impressed with nearly complete necrosis. Therefore, necrectomy of the necrotic tissue was performed revealing a tissue defect of the complete proximal third of the left palmar forearm. After a prolonged hospitalization coupled with several repetitive debridements, antibiotic therapy, as well as the immobilization of the left arm in a cast, the wound finally appeared vital and surgically clean. We decided to close the wound by performing a free parascapular flap to the left forearm (Figure 1 (Fig. 1)). Perioperatively, the patient received her regular prednisone dosage plus an extra-dosage during surgery. Both the flap and the wound healing were monitored carefully. After 5 days, a small dehiscence with serous outflow on the proximal wound occurred. After daily lavage and alginate wound dressing commenced, the wound healing was uncomplicated and finally, the patient could be discharged after a hospitalization period of nearly 10 weeks.

### Case 2

**Figure 2:**
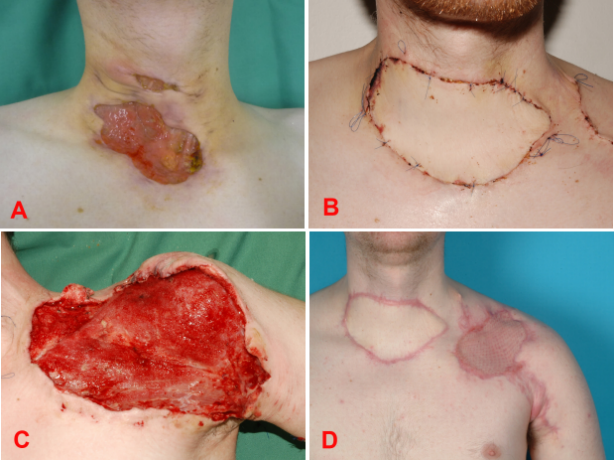
A 25-year-old male with a soft tissue defect due to an abscess after fault puncture of a central line (A); perfect wound healing of the supraclavicular island flap and at the elevation site before discharge (B); new wound defect after debridement at the elevation site 3 weeks after discharge (C); follow up 4 months after wound closure with Matriderm® and skin graft at the flap elevation site (D).

A 25-year-old male with colitis ulcerosa treated with prednisone was transferred to our department with a soft tissue defect at the left neck and upper left thorax after local necrosis following an abscess due to an erroneous puncture of a central line (Figure 2a (Fig. 2)). After several debridements, applying vacuum- and antibiotic therapy combined with continuing a low dose prednisone therapy, the wound was vital and microbiologically free of bacteria. As reconstructive surgery, we decided to perform a supraclavicular artery island flap. The postoperative wound healing process showed no signs of infection with only a minor dehiscence at the drainage area so that we could discharge the patient with a good reconstructive result (Figure 2b (Fig. 2)). 

However, following the necessity of increasing the prednisone therapy due to an acute period of colitis ulcerosa, the patient presented himself again 3 weeks after being discharged with multiple dehiscence around the elevation site of the supraclavicular flap with serous outflow. The microbiological tests showed no evidence of bacteria. After multiple debridements, the secondary wound was covered with skin grafts using MatriDerm^®^ as dermal matrix (Figure 2c, d (Fig. 2)). The young, immunocompromised patient could finally be discharged after an overall hospitalization stay of more than 12 weeks.

### Case 3

**Figure 3:**
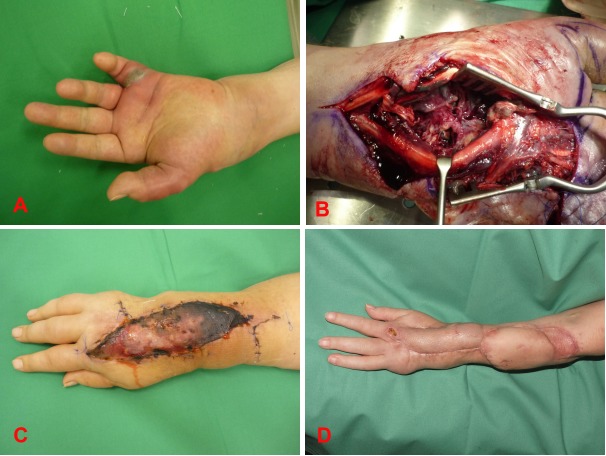
A 56-year-old female with a massive hand phlegmon due to an infected wrist prosthesis (A preoperatively, B intraoperatively); wound necrosis after reconstructive surgery with a free fibula flap (C); follow up result after several debridements and additional gracilis flap 5 months after discharge (D).

A 56-year-old female with a prosthetic left wrist implanted 8 years before due to chronic polyarthritis presented herself to our emergency department with a massive painful swelling and signs of hand phlegmon (Figure 3a (Fig. 3)). Her daily medication was methotrexate and prednisolone. Methrotrexate was paused, while prednisolone was reduced and the pain therapy adjusted by the pain therapist. We immediately performed a first debridement with relieving incisions. Daily wound lavage and antibiotic treatment were initiated. With no tangible improvement of the local wound signs and continuously increasing white blood cell count, we decided to remove the prosthetic wrist (Figure 3b (Fig. 3)), stabilizing the hand by external fixation. After two more debridements and wound closure, the patient could be discharged 4 weeks later with external fixation and ongoing low dose prednisolone. After full recovery, the patient was hospitalized again for the removal of the external fixation and for autologous wrist arthrodesis by performing a free fibula flap to the left wrist (Figure 3c (Fig. 3)). Once more, the wound healing was not adequate and both, staphylococcus and enteroccus could be verified within the wound. Antibiotic therapy was adjusted and additionally, we performed a gracilis flap to close the wound on the left wrist (Figure 3d (Fig. 3)). However, the elevation area of the flap on the lower limb showed wound healing problems requiring a debridement and local skin grafting for wound closure. 

With an overall hospitalization of more than 14 weeks, we could finally discharge the patient. 6 months after the last procedure was performed, again the patient was transferred to our hospital with the status of an acute sepsis and encephalitis with the left wrist being the focus of infection. As life-saving procedure the left hand needed to be amputated at the distal forearm. Finally, the patient recovered from both sepsis and encephalitis and could be discharged from our clinic to rehab. 

### Case 4

**Figure 4:**
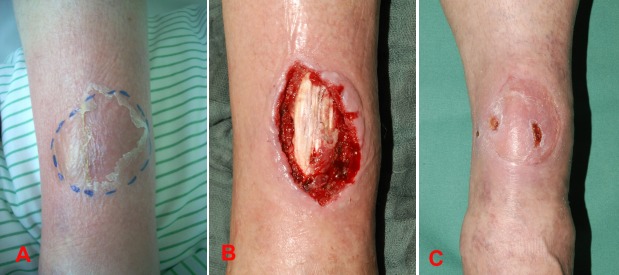
A 60-year-old female with an abscess and surrounding erysipelas on the left lower leg (A); wound defect size after first debridement (B); final result after vacuum therapy for improving wound granulation and skin grafting for defect closure (C).

A 60-year-old female with the initial diagnosis of acute myelogenous leukemia was presented to our clinic with an abscess and surrounding erysipelas on the left lower leg (Figure 4a (Fig. 4)). A first excision and debridement of the soft tissue was performed exposing the extensor tendons with a defect size of 6x5 cm (Figure 4b (Fig. 4)). Vacuum therapy was applied. Due to the initial and ongoing chemotherapy (idarubicin and cytosin-arabinoside) with a depleted hematological cell status, we decided not to perform a reconstructive procedure with a loco-regional or even free flap, but to repeat the debridement and vacuum therapy for the following 10 weeks. With a satisfying granulation of the wound, we finally closed the defect with skin grafts during a chemotherapeutic interval (Figure 4c (Fig. 4)). Although, the wound healing was prolonged, the patient could be discharged with finally good results to receive further allogenic stem cell transplantation in an external clinic.

### Case 5

A 72-year-old woman with the initial diagnosis of a malignant melanoma on the right lower leg presented to our clinic after an in sano excision in a clinic alio loco. However, after further excision of the melanoma, we closed the wound using skin mesh-graft. After complete wound healing, the patient received a low-dose interferon therapy with Roferon. After 3 months, the patients presented to us with worsening of the wound condition and a reappearance of activated melanoma in situ of the scar on the right lower leg. Therefore, a radical excision of the pretibial scar and a temporary covering with alloplastic material was performed. After several debridements, a clean granulating wound could be achieved making it possible to cover the defect of the lower leg with a free radial forearm flap from the right forearm. Finally, the patient could be discharged with normal wound healing from our department and received further chemotherapy without any sign of re-appearance of the malignant melanoma so far.

### Case 6

A 46-year-old male was presented to us with the fatal diagnosis of Fournier’s gangrene after receiving his first episodes of chemotherapy comprising of vincristine and prednisone due to a non-Hodgkin lymphoma. After an immediate debridement of the necrotic scrotal area reconstructive surgery was performed already two days later with loco-regional flaps from both sides. With good wound healing and stabilized overall condition, the patient could be discharged to rehab after being hospitalized for more than 12 weeks. 

### Case 7

A 57-year-old male patient with the initial diagnosis of an advanced malignant melanoma on the left foot was operated in our department with an excision in sano of the tumor and closing of the defect by performing a free anterolateral thigh flap (ALTP). However, diagnostic lymph node biopsy was performed as well and following positive histopathology findings, a groin dissection, which was performed without complications, was necessary. The vascular femoral structures were covered by a transposition of the sartorius muscle. As a matter of fact, the patient could be discharged within five days after an uncomplicated wound healing. 

Over the course of time, chemotherapy and radiotherapy was conducted on the affected groin by the department of dermatology. Unfortunately, positive signs of lymph metastasis were detected in the groin region again, thereby requiring the necessity of another operation for the removal of all stationary lymph nodes and subcutaneous tissue. This time wound healing was prolonged due to the immunocompromised status of the patient and the impaired skin in the groin after radiotherapy. With the formation of a chronic seroma regular puncture was necessary, which led to chronic lympho-edema, recurring erysipelas and a frequent re-hospitalisation of the patient. Finally, we decided together with the patient to re-operate the radiated groin. After several debridements the defect was closed by performing loco-regional advancement flaps. The final wound healing was acceptable with only a small dehiscence and no further seroma so far. Chemotherapy was continued by the department of dermatology after the patient had been discharged.

### Case 8

A 53-years-old male patient was presented to our department with a massive hand phlegmon arising from a small skin lesion a week earlier. Despite the swelling, redness and pain, necrotic areas were also present. However, due to chronic hepatitis C the patient was immunocompromised with interferon and ribavirin therapy. After performing a first debridement with necrectomy of the affected skin and soft tissue, vacuum therapy was applied. After several debridements with finally sufficient granulation, we decided to cover the defect by performing a radial forearm flap. This procedure could be successfully done after another period of vacuum therapy as the patient suffered from ascites and his general health conditions worsened due to his liver cirrhosis. The patient could finally be discharged after 7 weeks with good wound healing and recovery from the ascites.

## Results

In this retrospective analysis of immunocompromised patients, we directly performed reconstructive surgery after an initial debridement only in one patient. In this case of a Fournier’s gangrene in a middle-aged patient and the diagnosis of a non-Hodgkin lymphoma, the wound closure was supposed to be done urgently as further chemotherapy was planned contemporarily after wound healing. Fortunately, there was no sign of wound complications after the locoregional flaps were performed. 

However, in seven of our eight presented cases, several debridements have been necessary to optimize the wound granulation and to bridge the vulnerable time during immunosuppressive therapy. Thus, in all of these cases, the optimum time for reconstructive surgery had to be defined individually depending on the wound conditions, microbiological findings as well as immunosuppressive therapy and overall health status. Three of our patients needed further surgical treatment due to wound dehiscence or necrosis, with two of these patients as a result of increased immunosuppressive therapy after initial discharge from our hospital. 

One patient with chronic polyarthritis and immunocompromising therapy for more than 15 years needed several reconstructive procedures until the wound on the left hand healed completely. Unfortunately, six months after being discharged, the same patient underwent an amputation of her left hand after developing acute sepsis and encephalitis with the operated wrist being the focus of infection. 

In three of our cases we decided together with the patients and in consultation with the treating internal medicine department to accomplish the wound closing not by advanced flap surgery but with skin grafting to minimize possible postoperative complications and therefore a delay of further needed immunosuppressive treatment.

## Discussion

Reconstructive surgery of immunocompromised patients requires several steps of periooperative and surgical treatment. Unlike ‘normal’ patients, immunocompromised patients have increased risks of perioperative complications as a result of their disease and medical treatment [12]. 

Thus, corticosteroids are potent anti-inflammatory agents and are effective as therapeutic immunosuppressive therapy of different diseases such as Crohn’s disease or in transplant and tumor patients [3], [4], [5], [6], [7], [8], [9], [10], [11], [12], [13], [14]. However, despite many side effects Oxlund et al. already described in 1979 their findings in an in vivo rat model showing that moderate cortisol treatment resulted in impaired mechanical properties and reduced ability of the wounded skin to withstand rupturing forces [1]. Furthermore, corticosteroids can inhibit fibroplasia, vascular proliferation and wound contraction in soft tissue healing [15], [16]. As in many cases suspension of corticosteroid therapy is not possible due to the treated disease, it is recommended to reduce it to the lowest possible dosage during perioperative treatment [16]. In our presented cases, three of eight patients were treated with prednisone, which was reduced prior to surgery. However, in two of these patients, the dosage was increased by the primary care physician after the patients were discharged leading to serious wound complications and rehospitalization for further operative treatment.

Methotrexate (MTX) is commonly used in patients with rheumatoid arthritis and is a frequent component of chemotherapeutic regimens for a variety of tumor types [16], [17], [18]. Cohen et al. demonstrated a dose-dependent transient decrease in wound tensile strength when administered preoperatively [18]. Loza et al. showed that low doses of MTX seems to be a safe option during the perioperative period in patients with rheumatoid arthritis without relevant comorbidities or risk factors [17]. In our reported patients, only one patient was treated with MTX due to chronic polyarthritis. MTX was paused preceding the first surgery and reconvened two weeks after the last reconstructive procedure. However, a negative effect of the MTX treatment for several years prior to our surgical treatment cannot be ruled out. 

Furthermore, the acute phase of wound healing is partially triggered by activation of platelets through the release of platelet-derived growth factors (PDGF). PDGF is responsible for chemotactic and mitogenic effects on fibroblasts, smooth muscle cells, macrophages, monocytes and neutrophils [18]. Therefore, the depleted number of platelets and overall cell status in patients with ongoing chemotherapy is highly limiting the activation of wound healing. Six of our eight patients had ongoing chemotherapy or chemotherapy directly before being transferred to our department. In one case of massive cell depletion we decided to perform skin grafting instead of local flap surgery during an interval of chemotherapy with a good final result. In the other five cases, different kinds of loco-regional and free flaps were performed with wound healing complications in only one of them. However, this patient was not only treated with chemotherapy but also with radiotherapy prior to the elective surgery and had therefore an even more worsened precondition for operative procedures with a known significant increase in wound complications [19], [20].

In conclusion, the immunocompromised patient needs a more careful and more individually customized surgical and perioperative treatment. Furthermore, a continuous follow-up is necessary to realize possible complications and to adjust the medical treatment, especially concerning corticosteroid, to avoid late wound healing complications. 

Finally, reconstructive surgery in immunocompromised patients should be as simple as possible to reduce the peri- and postoperative risks and therefore to avoid unnecessary prolonged hospitalization.

## Notes

### Competing interests

The authors declare that they have no competing interests.

## Figures and Tables

**Table 1:**
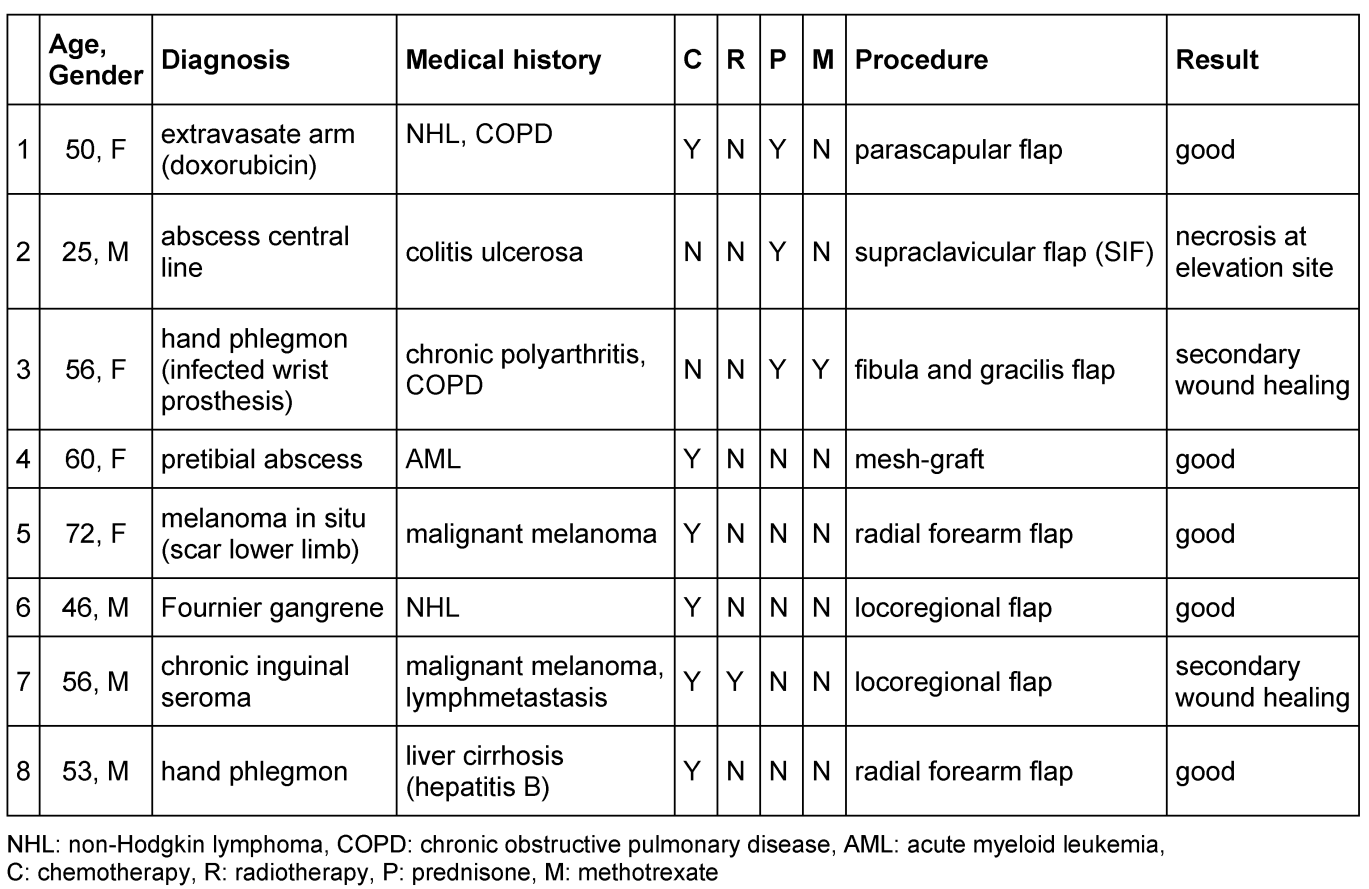
Immunocompromised patients undergoing reconstructive surgery

## References

[1] Oxlund H, Fogdestam I, Viidik A (1979). The influence of cortisol on wound healing of the skin and distant connective tissue response. Surg Gynecol Obstet.

[2] Graeb C, Jauch KW (2008). Surgery in immunocompromised patients. Br J Surg.

[3] Papadopoulos O, Konofaos P, Chrisostomidis C, Lionaki S, Georgiou P, Vlasis K, Kostakis A (2005). Reconstructive surgery for kidney transplant recipients. Transplant Proc.

[4] Qi FZ, Zhang Y, Yang Z, Feng ZH, Gu JY (2009). Plastic surgery after solid organ transplantations. Chin Med J.

[5] Grisafi JL, Dadachanji C, Rahbar R, Detschelt E, Benckart DH, Muluk SC (2011). The effect of immunosuppression on lower extremity arterial bypass outcomes. Ann Vasc Surg.

[6] Cohen M, Pollak R, Garcia J, Mozes MF (1989). Reconstructive surgery for immunosuppressed organ-transplant recipients. Plast Reconstr Surg.

[7] Armstrong MB, Villalobos RE, Leppink DM (1997). Free-tissue transfer for lower-extremity reconstruction in the immunosuppressed diabetic transplant recipient. J Reconstr Microsurg.

[8] Sbitany H, Xu X, Hansen SL, Young DM, Hoffman WY (2014). The effects of immunosuppressive medications on outcomes in microvascular free tissue transfer. Plast Reconstr Surg.

[9] Miller MW, Dean NR, Cannady SB, Rosenthal EL, Wax MK (2012). Free tissue transfer for head and neck reconstruction in solid organ transplant patients. Head Neck.

[10] Knight RJ, Villa M, Laskey R, Benavides C, Schoenberg L, Welsh M, Kerman RH, Podder H, Van Buren CT, Katz SM, Kahan BD (2007). Risk factors for impaired wound healing in sirolimus-treated renal transplant recipients. Clin Transplant.

[11] Lee AB, Dupin CL, Colen L, Jones NF, May JW, Chiu ES (2008). Microvascular free tissue transfer in organ transplantation patients: is it safe?. Plast Reconstr Surg.

[12] Chang J, Davis CL, Mathes DW (2012). The impact of current immunosuppression strategies in renal transplantation on the field of reconstructive transplantation. J Reconstr Microsurg.

[13] Irving PM, Gearry RB, Sparrow MP, Gibson PR (2007). Review article: appropriate use of corticosteroids in Crohn's disease. Aliment Pharmacol Ther.

[14] Green JP (1965). Steroid therapy and wound healing in surgical patients. Br J Surg.

[15] Hatz RA, Kelley SF, Ehrlich HP (1989). The tetrachlorodecaoxygen complex reverses the effect of cortisone on wound healing. Plast Reconstr Surg.

[16] Busti AJ, Hooper JS, Amaya CJ, Kazi S (2005). Effects of perioperative antiinflammatory and immunomodulating therapy on surgical wound healing. Pharmacotherapy.

[17] Loza E, Martinez-Lopez JA, Carmona L (2009). A systematic review on the optimum management of the use of methotrexate in rheumatoid arthritis patients in the perioperative period to minimize perioperative morbidity and maintain disease control. Clin Exp Rheumatol.

[18] Cohen SC, Gabelnick HL, Johnson RK, Goldin A (1975). Effects of antineoplastic agents on wound healing in mice. Surgery.

[19] Mushtaq S, Goodman SM, Scanzello CR (2011). Perioperative management of biologic agents used in treatment of rheumatoid arthritis. Am J Ther.

[20] Payne WG, Naidu DK, Wheeler CK, Barkoe D, Mentis M, Salas RE, Smith DJ DJ, Robson MC (2008). Wound healing in patients with cancer. Eplasty.

